# MAIT Cells Balance the Requirements for Immune Tolerance and Anti-Microbial Defense During Pregnancy

**DOI:** 10.3389/fimmu.2021.718168

**Published:** 2021-08-09

**Authors:** Johanna Raffetseder, Robert Lindau, Sigrid van der Veen, Göran Berg, Marie Larsson, Jan Ernerudh

**Affiliations:** ^1^Division of Inflammation and Infection (II), Department of Biomedical and Clinical Sciences (BKV), Linköping University, Linköping, Sweden; ^2^Division of Obstetrics and Gynecology, and Department of Biomedical and Clinical Sciences (BKV), Linköping University, Linköping, Sweden; ^3^Division of Molecular Medicine and Virology (MMV), Department of Biomedical and Clinical Sciences (BKV), Linköping University, Linköping, Sweden; ^4^Department of Clinical Immunology and Transfusion Medicine, and Department of Biomedical and Clinical Sciences, Linköping University, Linköping, Sweden

**Keywords:** mucosal-associated invariant T (MAIT) cells, pregnancy, innate-like T cells, decidua, T cells, immune tolerance, anti-microbial response, immunomodulation

## Abstract

Mucosal-associated invariant T (MAIT) cells are an innate-like T cell subset with proinflammatory and cytotoxic effector functions. During pregnancy, modulation of the maternal immune system, both at the fetal-maternal interface and systemically, is crucial for a successful outcome and manifests through controlled enhancement of innate and dampening of adaptive responses. Still, immune defenses need to efficiently protect both the mother and the fetus from infection. So far, it is unknown whether MAIT cells are subjected to immunomodulation during pregnancy, and characterization of decidual MAIT cells as well as their functional responses during pregnancy are mainly lacking. We here characterized the presence and phenotype of Vα7.2^+^CD161^+^ MAIT cells in blood and decidua (the uterine endometrium during pregnancy) from women pregnant in the 1^st^ trimester, *i.e.*, the time point when local immune tolerance develops. We also assessed the phenotype and functional responses of MAIT cells in blood of women pregnant in the 3^rd^ trimester, *i.e.*, when systemic immunomodulation is most pronounced. Multi-color flow cytometry panels included markers for MAIT subsets, and markers of activation (CD69, HLA-DR, Granzyme B) and immunoregulation (PD-1, CTLA-4). MAIT cells were numerically decreased at the fetal-maternal interface and showed, similar to other T cells in the decidua, increased expression of immune checkpoint markers compared with MAIT cells in blood. During the 3^rd^ trimester, circulating MAIT cells showed a higher expression of CD69 and CD56, and their functional responses to inflammatory (activating anti-CD3/CD28 antibodies, and IL-12 and IL-18) and microbial stimuli (*Escherichia coli*, group B streptococci and influenza A virus) were generally increased compared with MAIT cells from non-pregnant women, indicating enhanced antimicrobial defenses during pregnancy. Taken together, our findings indicate dual roles for MAIT cells during pregnancy, with an evidently well-adapted ability to balance the requirements of immune tolerance in parallel with maintained antimicrobial defenses. Since MAIT cells are easily activated, they need to be strictly regulated during pregnancy, and failure to do so could contribute to pregnancy complications.

## Introduction

Pregnancy poses a unique challenge to the maternal immune system due to the semi-allogeneic nature of the fetus. Local immune adaptations occur early during pregnancy and serve to create a tolerant environment at the fetal-maternal interface to allow for successful implantation and tissue remodeling. The decidua, the maternal part of the fetal-maternal interface, is characterized by the presence of tissue homeostatic, M2-like macrophages, regulatory NK cells, and regulatory T cells, while other T cells as well as B cells are mainly excluded from this highly specialized environment (1, 2).

Systemically, immune regulation during pregnancy has been described as a shift of the Th1/Th2-balance towards a Th2-skewing of immune responses (3). However, other studies indicate that this is an oversimplification and suggest a general dampening of adaptive responses (3, 4). Furthermore, the numbers and activity of innate immune cells (monocytes, dendritic cells and neutrophils) were found to increase, while lymphocytes decreased (1, 4, 5). These systemic immune alterations could explain clinical phenomena observed during pregnancy: Firstly, pregnant women, especially during the 3^rd^ trimester, are more susceptible to infections such as *Listeria monocytogenes*, influenza and herpes simplex virus, which are typically dependent on Th1 responses. There is also an increased morbidity and mortality of certain viral diseases, in particular influenza A infection (3, 5). Secondly, pregnant women with autoimmune diseases such as multiple sclerosis and rheumatoid arthritis experience a significant alleviation of symptoms and a transient decrease in relapse rate during the 3^rd^ trimester (6, 7). Combined, these observations demonstrate that the maternal immune system undergoes immunomodulation during pregnancy, not only at the fetal-maternal interface but also at a systemic level.

Mucosal-associated invariant T (MAIT) cells are an innate-like T cell-subset with proinflammatory and cytotoxic effector functions. They make up a substantial portion of T cells at mucosal sites but are most abundant in the liver. In the circulation they make up 0.1-10% of the T cells (8). MAIT cells carry a semi-invariable T cell receptor (TCR) consisting of the Vα7.2 (TRAV1-2) segment in the variable region of the α-chain and one of three possible segments in the joining region (Jα33, Jα12, or Jα20) (9, 10). In turn, this semi-invariable α-chain is paired with a limited number of β-chains. This TCR is restricted to the MHC class I-like molecule (MR1) (10), which is structurally similar to conventional MHC class I molecules. As opposed to conventional MHC molecules, no polymorphisms are known for MR1, and MR1 is highly conserved among mammals (11). MR1 presents small molecules, namely from the riboflavin (vitamin B2) and folic acid (vitamin B9) biosynthesis pathways (12, 13). Most bacteria, but also yeasts are capable of riboflavin biosynthesis, and therefore MAIT cells typically recognize and are activated by cells infected with these microorganisms (14). Located primarily in mucosal tissues, MAIT cells have intrinsic effector capacity and readily react to activation without the need of prior activation and clonal expansion in lymphatic tissues, in similarity to innate immune cells. Upon activation, MAIT cells secrete Th1 and Th17-cytokines (TNF, IFN*γ*, IL-17 and IL-22), but also cytotoxic effector molecules (granzyme B and perforin). In addition to the TCR, MAIT cells carry receptors for type I IFNs, IL-12, IL-15 and IL-18, and can therefore also be activated by proinflammatory cytokines (15, 16), for example during viral infections or sterile inflammation, thereby utilizing a MR1/TCR-independent route of activation. Since MAIT cells can respond readily to antigens and proinflammatory cytokines, and due to the highly conserved MR1 and the limited TCR variation, MAIT cells constitute a first line of defense against infections at mucosal sites. Although MAIT cells are quick responders to inflammation, in line with the innate nature of MAIT cells, they have also been described to be involved in tissue homeostasis and tissue repair (8). Similar to conventional T cells, MAIT cells can express the activation marker HLA-DR and the immune checkpoint markers PD-1 and CTLA-4 (8). The function of HLA-DR on T cells, other than serving as an activation marker, is still elusive, while the checkpoint inhibitors PD-1 and CTLA-4 dampen T cell responses *via* co-inhibitory signaling, presumably including responses in MAIT cells (17).

In line with the different functions of MAIT cells, phenotypic heterogeneity has been described at the cellular level. Most MAIT cells in blood are CD8^+^ while minor CD4^+^ and double negative (DN) subsets are also present (18, 19). In several mucosal tissues increased proportions of DN MAIT cells have been reported, with possible functional implications (8, 20). Furthermore, the CD56^+^ subset of MAIT cells has been shown to mount stronger responses to innate cytokines (21).

Regarding their localization in mucosal tissues with reproductive relevance, MAIT cells have been shown to be present in the female genital mucosa, and these tissue-resident MAIT cells were phenotypically altered (20) as they responded with production of IL-17 and IL-22 to bacterial stimulation, while circulating MAIT cells produced IFN*γ*, TNF and granzyme B (20). In contrast, MAIT cells isolated from term decidua responded to bacterial challenge with production of IFN*γ*, granzyme B and perforin at a similar magnitude as MAIT cells in peripheral blood (22). Furthermore, MAIT cells were found to be retained in the intervillous blood in term placenta; these cells responded to stimulation with the production of IFN*γ* and cytotoxic effector molecules and could serve to defend the fetal-maternal interface from bacterial infections (22–24). In contrast, very little is known about MAIT cells in the early stage of pregnancy. They are known to be present in the 1^st^ trimester decidua (25, 26), but a detailed characterization of decidual MAIT cell frequencies and phenotypes is lacking. Furthermore, it is not known if functional responses of circulating MAIT cells are altered during pregnancy compared with the non-pregnant state. Since pregnant women have an increased risk for more severe infections during the 3^rd^ trimester of pregnancy (5, 27) and since vaginal colonization with certain bacteria is associated with increased risk of preterm birth (28), it is relevant to assess the functional responses of MAIT cells in relation to microbial stimuli.

Taken together, innate immune cells and functions are more pronounced during pregnancy, both at the fetal-maternal interface and systemically. However, so far very little is known about the innate-like MAIT cells and their presence and functional involvement during pregnancy*, i.e*., if they are adapted to the state of immune tolerance and if they retain their innate properties of rapidly responding anti-microbial cells. We therefore assessed the frequency and phenotypes of MAIT cells at the fetal-maternal interface during the 1^st^ trimester of pregnancy, when local immunomodulation is developing, and systemically during the 3^rd^ trimester, when systemic immunomodulation is most pronounced. We report that MAIT cells – being proinflammatory and highly reactive cells – are relatively excluded from the fetal-maternal interface and that remaining cells are adapted to immune tolerance by expression of markers of immunomodulation. Furthermore, we report that responses of circulating MAIT cells to microbial and inflammatory stimuli are increased during pregnancy, likely as part of an increased innate response that compensates for the decreased responses of conventional T cells. Being described as innate-like T cells, our findings indicate that MAIT cells balance the dual requirements during pregnancy: in the decidua, MAIT cells are regulated in similarity to conventional T cells, while circulating MAIT cells show enhanced functional responses similar to innate immune cells.

## Materials and Methods

### Subjects and Sample Collection

For the analysis of MAIT cells at the fetal-maternal interface, 1^st^ trimester decidual tissues and paired venous blood samples were collected from 24 healthy pregnant women undergoing elective surgical abortions at the Women’s Clinic at Linköping University Hospital. All pregnancies were viable as determined by ultrasound. The median gestational week was 10+2 as determined by crown-rump length by ultrasound. All women received misoprostol (Cytotec) prior to the surgery. For details on study participants and their history of previous pregnancies, see **Table 1**.

**Table 1:** Characteristics of study participants. n.a. not applicable.

	Non-pregnant	Pregnant
**1^st^ trimester**		
Number of women	n.a.	24
Age; median (range)	n.a.	28.0 (19-39)
Gestational age at timepoint of surgery; median (range)	n.a.	10+2 (8+0 – 11+4)
Number of previous pregnancies; median (range)	n.a.	2 (0-8)
Number of previous births; median (range)	n.a.	1 (0-4)
**3^rd^ trimester**		
Number of women	26	26
Age; median (range)^1^	27.2 (22-33)	28.6 (19-35)
Gestational age at blood sampling, median (range)	n.a.	35+4 (34+0 – 36+6)
Gestational week of delivery	n.a.	40.5 (38-43)
Sex of newborn; female/male	n.a.	13/13
Number of previous pregnancies; median (range)^1^	0 (0-1)	1 (0-5)
Number of previous births; median (range)^1^	0 (0-1)	1 (0-4)
Medication at blood sampling; number of women (%)		
Oral contraceptives	9 (34.6%)	n.a.
Inhalation budesonide and inhalation terbutaline sulfate^2^	1 (3.8%)	0 (0%)
Selective serotonin re-uptake inhibitors^2^	2 (7.7%)	2 (7.7%)
Low-dose acetylsalicylic acid^2^	0 (0%)	2 (7.7%)
Pain medication (paracetamol/codein)^2^	0 (0%)	3 (11.5%)

^1^Mann-Whitney test was used to test for statistical differences in age (not significant), number of previous pregnancies (p < 0.0001) and number of previous births (p < 0.0001).

^2^Fisher’s exact test was used to test for differences in medication at blood sampling (not significant for any of the medications). n.a., not applicable.

For the analysis of circulating MAIT cells during the 3^rd^ trimester of pregnancy, blood samples were collected from healthy 3^rd^ trimester pregnant women (n=26) and age-matched, non-pregnant healthy female controls (n=26) at the Vrinnevi Hospital Norrköping and at Linköping University Hospital. Regarding the pregnant women, all pregnancies were uncomplicated, and samples were taken at median gestational age 35+4 during a routine checkup at the maternal ward. Non-pregnant controls were recruited among students and personnel at Linköping University and Linköping University Hospital. All non-pregnant women were healthy. For details on study participants, see **Table 1**.

The studies were approved by the regional ethics review board in Linköping (M 39-08 and 2018/69-32), and inclusion and sampling occurred after informed written consent.

### Isolation of Cells

Venous blood was collected in sodium heparin tubes (Greiner Bio-One). Peripheral blood mononuclear cells (PBMCs) were isolated by gradient centrifugation using Lymphoprep (Axis-Shield). Freshly isolated PBMCs were used for phenotyping of cells by flow cytometry. From the 3^rd^ trimester pregnant women and healthy controls, PBMCs were also frozen for *in vitro* stimulation assays using 10% dimethylsulfoxide in 50% fetal calf serum (FCS, HyClone, Cytiva Life Sciences) and 40% Roswell Park Memorial Institute (RPMI) 1640 medium.

For the isolation of decidual cells, a previously published protocol was adapted (29). Decidual tissues were taken care of immediately after surgery. Tissues were rinsed with saline solution and placed in cold Iscove’s modified Dulbecco’s medium (Gibco, Thermo Fisher), supplemented with 292 µg/ml L-glutamine (Sigma-Aldrich), 3.024 mg/ml sodium bicarbonate (Sigma-Aldrich), 50 U/ml penicillin and 50 μg/ml streptomycin (Penicillin-Streptomycin Mixture, Lonza Bioscience) and 1x minimal essential medium non-essential amino acids (Gibco, Thermo Fisher). Tissues were kept on ice until cells were isolated. To do so, tissue was rinsed with PBS, blood clots were removed, and the tissue cut into smaller pieces, before being subjected to enzymatic digest with 255 U/ml collagenase IV (Worthington) and 40 U/ml DNase I (Sigma Aldrich) diluted in RPMI 1640 medium, supplemented with 5% FCS (HyClone, Cytiva Life Sciences) and 100 U/ml penicillin, 100 µg/ml streptomycin and 292 µg/ml L-glutamine (Penicillin-Streptomycin-Glutamine, Gibco, Thermo Fisher).

For enzymatic digest, the tissue pieces were incubated for 20 min at room temperature with vigorous shaking, followed by centrifugation. This procedure was repeated two times with fresh digest medium, followed by filtering of the cell suspension and lysis of red blood cells (BD Pharm Lyse, Becton Dickinson). Before antibody staining for flow cytometry, cells were counted.

### Functional Experiments

For *in vitro* stimulations, frozen PBMCs from 3^rd^ trimester pregnant women (n=14) and non-pregnant controls (n=14) were used. Cells were thawed, resuspended in prewarmed RPMI medium consisting of RPMI 1640 medium supplemented with 10% FCS (HyClone, Cytiva Life Sciences), and 50 U/ml penicillin and 50 μg/ml streptomycin (Penicillin-Streptomycin Mixture, Lonza Bioscience) and washed three times in the same medium. Cells and stimulating agents were diluted in the same medium. 500 000 PBMCs per well were used for stimulations.

For stimulation with anti-CD3 and anti-CD28 antibodies (both from Biorad), flat-bottom 96-well plates (Corning) were coated the day before stimulation. Antibodies were diluted in cold PBS, pooled, and further diluted to a final concentration of 0.3 µg/ml each. 100 µl of this solution were used per well, and 100 µl PBS were added to the wells for the unstimulated samples. Plates were incubated at 4 °C overnight. After incubation and before addition of cells, unbound antibodies were washed away by three washes with PBS.

For the generation of fixed *Escherichia coli*, the DH5α strain was grown overnight in LB-Lennox medium, washed with PBS, and fixed with 1% paraformaldehyde (Sigma Aldrich) for 20 min, followed by three washes with PBS. Before fixation, a small sample was taken and plated on LB plates to estimate bacterial numbers by determination of colony forming units (CFU). For the generation of group B streptococci (GBS, hemolytic strain, clinical isolate, Linköping University), the same procedure was carried out, except that bacteria were cultured in tryptic soy broth, and CFU plating was done on tryptic soy agar plates. Fixed bacterial stocks were aliquoted and stored frozen. For stimulation of PBMCs with fixed bacteria, *E. coli* and GBS were washed with PBS by centrifugation before diluting in RPMI medium. PBMCs were stimulated with a multiplicity of infection (MOI) of 10 for *E. coli* and MOI 1 for GBS.

For stimulation with recombinant IL-12p70 (Peprotech) and IL-18 (Biolegend), cytokines were pooled and diluted in RPMI medium to a concentration of 30 ng/ml each.

For stimulation with influenza A virus, the H1N1 strain A/PR/8/34 (purified antigen, obtained from Charles River) was added to PBMCs at a titer of 25 HA/ml.

Unstimulated cells were used as controls. Cells were incubated for 20 hours in total and GolgiPlug (BD Cytofix/Cytoperm kit, Becton Dickinson) diluted 1:1000 was added for the last 4 hours of incubation before cells were stained for flow cytometric analysis.

Stimulations were optimized on PBMCs from non-pregnant donors, and dose-dependent responses of MAIT cells were observed. For the stimulation of samples from the cohort, concentrations of stimuli were chosen that activated MAIT cells at an intermediate level, to allow for both increases and decreases in activation.

### Flow Cytometry

Decidual cells and PBMCs were stained for flow cytometric analysis using standard staining procedures. See **Supplementary Table S1** for antibodies used. Before staining with antibodies, all samples were stained with Live/Dead Fixable Aqua Dead cell stain (Molecular Probes, Thermo Fisher) for the exclusion of dead cells.

Cells were incubated with antibodies for surface markers, followed by fixation and permeabilization where indicated. For decidual samples and PBMCs from the same donors, the eBioscience™ Intracellular Fixation & Permeabilization Buffer Set (Thermo Fisher) was used for fixation and permeabilization prior to staining for intracellular markers. Stimulated PBMCs from women pregnant in the 3^rd^ trimester and from non-pregnant controls were fixed and permeabilized with Cytofix/Cytoperm (Becton Dickinson), followed by staining for intracellular proteins. Staining with the MR1 5-OP-RU and 6-FP tetramers was carried out at room temperature, before staining with antibodies for surface markers. Samples were acquired on an Aria III flow cytometer (Becton Dickinson) and all data was analyzed in Kaluza version 2.1 (Beckman Coulter). Gates were set either based on clear negative/positive populations or based on staining with isotype antibodies.

### Statistics

Because of non-normal distribution of most data sets, non-parametric statistical testing was performed. For comparisons between paired samples, *i.e.*, blood and decidua from the same woman, data were analyzed using Wilcoxon signed-rank test. For the comparison between the pregnant and non-pregnant group, Mann Whitney U test was used. Significance levels of p<0.05 were accepted as significant. All statistical analyses were performed in GraphPad Prism version 9 for Windows.

## Results

### MAIT Cells Are Present in 1^st^ Trimester Decidua at a Significantly Lower Proportion Than in Blood

**Figure 1:**
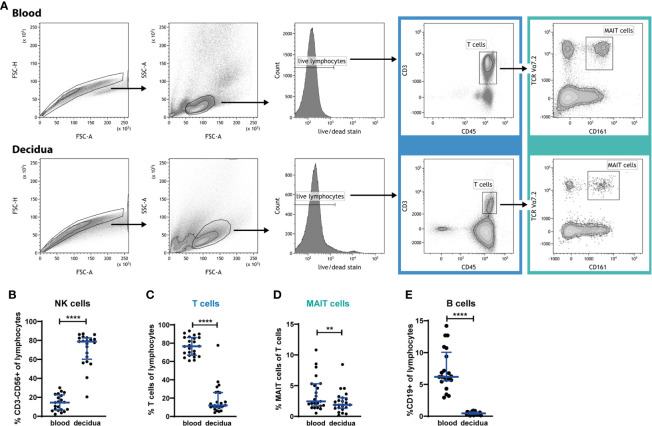
MAIT cells are present in 1^st^ trimester decidua, but are relatively excluded from the decidua. **(A)** Gating strategy for circulating (blood) and decidual T cells and MAIT cells. PBMCs and decidual cells were isolated from 1^st^ trimester blood and decidual tissue, respectively, from the same donors, and analyzed by flow cytometry. MAIT cells are identified by their expression of CD45, CD3, Vα7.2 and CD161. **(B)** NK cell, **(C)** T cell, **(D)** MAIT cell, and **(E)** B cell frequency in PBMCs (blood) and in cells isolated from decidua, expressed as percent of CD45^+^ lymphocytes, or – in the case of MAIT cells – expressed as percent of T cells. Gating strategies for NK and B cells are presented in **Supplementary Figure S2**. Bars depict median and interquartile range (IQR) from 21 donors in **(B, E)**, 24 donors in **(C)** and 23 donors in **(D)**. Statistical comparisons were performed with Wilcoxon signed-rank test. ** p < 0.01, **** p < 0.0001.

To better understand the role of MAIT cells at the fetal-maternal interface during early pregnancy we first enumerated the frequency of MAIT cells in 1^st^ trimester decidua tissues and related their frequency to the distribution in blood as well as to other lymphocyte populations. Decidual mononuclear cells and peripheral blood mononuclear cells (PBMCs) were analyzed by flow cytometry, and MAIT cells were defined by expression of the semi-invariant TCR chain Vα7.2 as well as the C-type lectin receptor CD161 (**Figure 1A**). Analysis with the MR1 tetramer (13) demonstrated that a large majority of the cells here defined as MAIT cells (Vα7.2^+^CD161^+^) also stain with the MR1 5-OP-RU tetramer (**Supplementary Figure S1**).

The distribution of decidual lymphocytes showed, in line with previous reports (1, 2), that NK cells are highly enriched in the decidua, while T cells are relatively excluded from this compartment (gating strategies for subpopulations of T cells, MAIT cells, NK cells and B cells are shown in **Supplementary Figure S2**). NK cells constituted 14% (median) of lymphocytes in the blood and 79% in the decidua (**Figure 1B**), while CD3^+^ T cells constituted 77% of lymphocytes in blood, but only 12% in decidua (**Figure 1C**). MAIT cells were also excluded from the decidua, with a significantly lower proportion within the T cell compartment (1.9% of all T cells) compared with blood (2.4% of all T cells, **Figure 1D**). Thus, MAIT cells are excluded to an even higher degree than the overall T cell population. MAIT cell frequencies, when expressed as a proportion of all CD45^+^ lymphocytes, showed that 0.2% in the decidua and 1.8% in blood are MAIT cells (data not shown), which implies that T cells were decreased 6.4-fold while MAIT cells are 9-fold lower in the decidua than in blood.

Furthermore, we analyzed the frequency of B cells, and found very low levels in the decidua; 0.5% of lymphocytes compared with 6.2% in the circulation (**Figure 1E**), which is in line with earlier reports (30–32). This almost complete absence of B cells, in combination with the typical composition of immune cells in our decidual samples, confirms that the decidual cells are not contaminated by blood cells from the highly vascularized decidual tissue.

### The Distribution of Decidual MAIT Cell Subsets Differs From MAIT Cell Subsets in Blood

**Figure 2:**
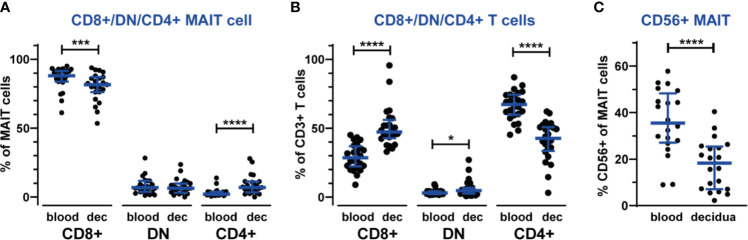
Different composition of MAIT cell subsets in the decidua compared with blood. Proportions of CD8^+^, CD4^+^ and double-negative (DN) subsets of MAIT cells **(A)** and non-MAIT T cells **(B)** in blood and decidua (dec). The gating strategy for CD8^+^, CD4^+^ and DN cells is shown in **Supplementary Figure S2**. MAIT cells were further analyzed for CD56 expression **(C)**. Gating of CD56^+^ MAIT is shown in **Supplementary Figure S3**. Bars show median and IQR from 20-24 donors. Statistical comparisons were performed with Wilcoxon signed-rank test. * p < 0.05, *** p < 0.001, **** p < 0.0001.

During pregnancy, the decidual lymphocytes are altered in terms not only of composition but also in terms of cellular subsets to create a tolerant environment. Therefore, in addition to establishing that MAIT cells are in part excluded from the decidua, we also investigated the expression of several phenotypic markers. The majority of circulating MAIT cells are CD8-positive, while also CD4-positive and double-negative (DN) MAIT cells have been reported (18, 19, 33). We therefore investigated the CD4/CD8-distribution of circulating and decidual MAIT cells. In accordance with earlier reports, most circulating MAIT cells were CD8^+^ (median 88%) and few were CD4^+^ (2.1%) or DN (6.8%). However, decidual MAIT cells exhibited a significantly skewed CD4/CD8-distribution, with a lower proportion of CD8^+^ (81%) and a higher proportion of CD4^+^ (6.9%) compared with their counterparts in blood (**Figure 2A**). The proportion of DN MAIT cells was unaltered compared with blood (3.7% in blood and 3.1% in decidua, **Figure 2A**). In contrast, but in line with previous reports (2), the total CD3^+^ T cell population in the decidua was skewed in the opposite direction, with a lower proportion of CD4^+^ (67% in blood and 43% in decidua) and higher proportions of CD8^+^ and DN T cells in decidua than in blood (CD8^+^: 29% in blood and 47% in decidua, DN T cells: 3.0% in blood and 4.7% in decidua, **Figure 2B**). The different distribution of CD4/CD8 expression suggests different functional properties of decidual MAIT cells compared with their counterparts in blood.

CD56^+^ MAIT cells have recently been established as a distinct subset of MAIT cells, exhibiting functional differences from CD56- MAIT cells such as increased expression of receptors for innate cytokines (21). The percentage of CD56^+^ MAIT cells in our blood samples are in the same range as reported earlier (26, 34, 35), while we detected fewer CD56^+^ MAIT cells in the decidua (35% in blood and 18% in decidua, **Figure 2C** and **Supplementary Figure S3**), which could reflect a mechanism to limit the numbers of CD56^+^ MAIT cells that are easily activated by proinflammatory cytokines in the decidua. We did not observe any discrete CD56 subpopulations based on level of CD56 expression, *i.e.*, no CD56^bright/dim^ subsets of MAIT cells.

### Decidual MAIT Cells Express Activation Markers and Markers of Immunoregulation

**Figure 3:**
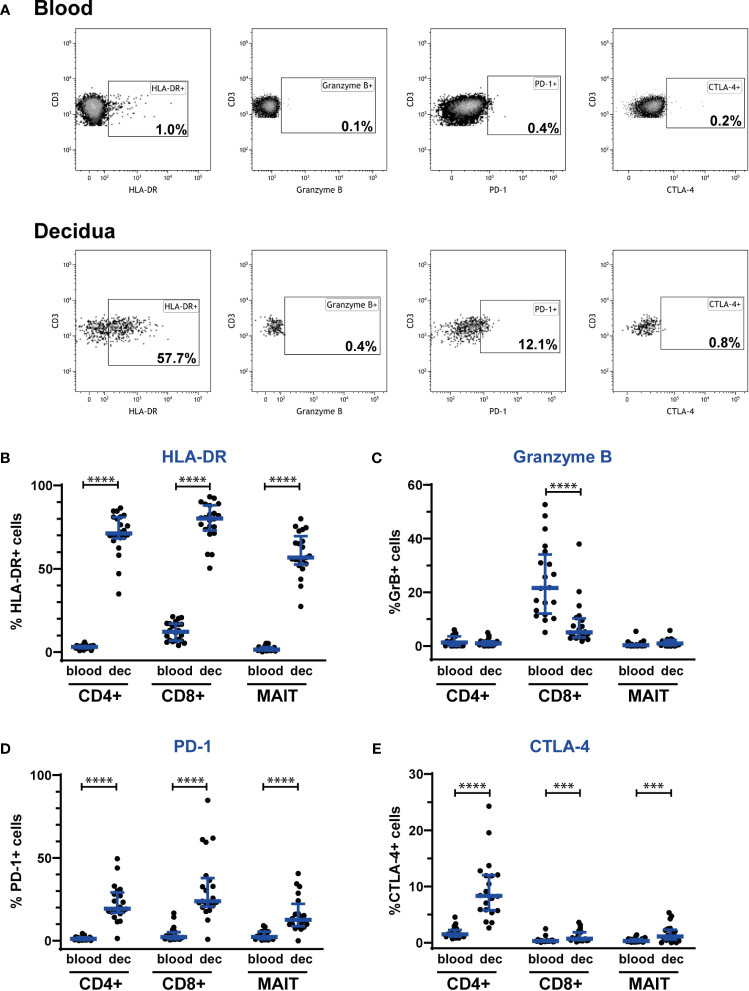
Decidual MAIT cells express activation and immune checkpoint markers and exhibit a fundamentally different phenotype than circulating MAIT cells from the same women. PBMCs and decidual cells were isolated from blood and decidual tissue (dec), respectively, from the same donors, and analyzed by flow cytometry. **(A)** illustrates the gating strategy employed for the phenotypic markers HLA-DR, granzyme B, PD-1, and CTLA-4 on Vα7.2^+^CD161^+^ MAIT cells for one representative blood and decidua sample. Positive gates for HLA-DR, granzyme B and PD-1 were set based on isotype controls, whereas the gate for CTLA-4 was set visually. Expression of **(B)** HLA-DR, **(C)** granzyme B, **(D)** PD-1 and **(E)** CTLA-4 by CD4^+^ T cells (CD4+), CD8^+^ T cells (CD8^+^) and MAIT cells (MAIT). Results for HLA-DR and PD-1 were based on cell surface staining, whereas granzyme B and CTLA-4 were stained intracellularly. From the CD4^+^ and CD8^+^ T cells, Vα7.2^+^CD161^+^ cells were excluded, *i.e.*, the CD4^+^ and CD8^+^ T cell populations do not comprise any MAIT cells. Results for NK cells based on the same samples are shown in **Supplementary Figure S4**. Bars show median and IQR from 20-22 donors. Statistical comparisons were performed with Wilcoxon signed-rank test. *** p < 0.001, **** p < 0.0001.

To further evaluate the phenotype of decidual MAIT cells, we investigated the presence of the activation marker HLA-DR and the cytolytic marker granzyme B. We observed a profound increase in HLA-DR surface expression on CD4^+^, CD8^+^, NK and MAIT cells in the decidua, as compared with blood (**Figures 3A, B**, and **Supplementary Figure S4**). Once activated, MAIT cells can produce cytotoxic effector molecules such as granzyme B (8). Notably, granzyme B showed no or very low expression on MAIT cells, both in the decidua and in blood, as was also the case for CD4^+^ T cells. In contrast, CD8^+^ T cells and NK cells showed a high expression of granzyme B, albeit significantly lower in the decidua compared with blood (**Figures 3A, C**, and **Supplementary Figure S4**). Thus, MAIT cells in the decidua exhibit an activated (HLA-DR^+^) but disarmed (granzyme B^low^) phenotype in relation to cytotoxic function.

Finally, to reveal any immunomodulatory potential of MAIT cells, we investigated the expression of the immune checkpoint markers PD-1 and CTLA-4. Interestingly, MAIT cells, as well as CD4^+^ and CD8^+^ T cells showed an increased expression of PD-1 and CTLA-4 in the decidua compared with blood (**Figures 3A, D, E**). Decidual NK cells expressed more CTLA-4, but surface expression of PD-1 was unaltered compared with blood (**Supplementary Figure S4**).

Taken together, MAIT cells in the decidua exhibit a disarmed CD56^low^/granzyme B^low^ phenotype with a coinciding increased expression of immune checkpoint markers, strongly suggestive of an adaptation to the immune-tolerant environment at the fetal-maternal interface, aiming to achieve a default restriction of cytotoxicity and unwanted T cell activation.

### Circulating MAIT Cells Are Present at Unaltered Frequency During the 3^rd^ Trimester of Pregnancy, but Show a More Activated Phenotype

Having established that decidual MAIT cells are subjected to immunomodulation early during pregnancy, when local tolerance is developing, we proceeded to characterize circulating MAIT cells during the 3^rd^ trimester, when systemic immunomodulation is most pronounced (5–7). To do so, we collected blood samples from women with uncomplicated pregnancies in median gestational week 35+4 as well as from non-pregnant controls (n=26 in each group). The non-pregnant controls were age-matched, since MAIT cell numbers have been suggested to decline during aging (33, 36–38).

**Figure 4:**
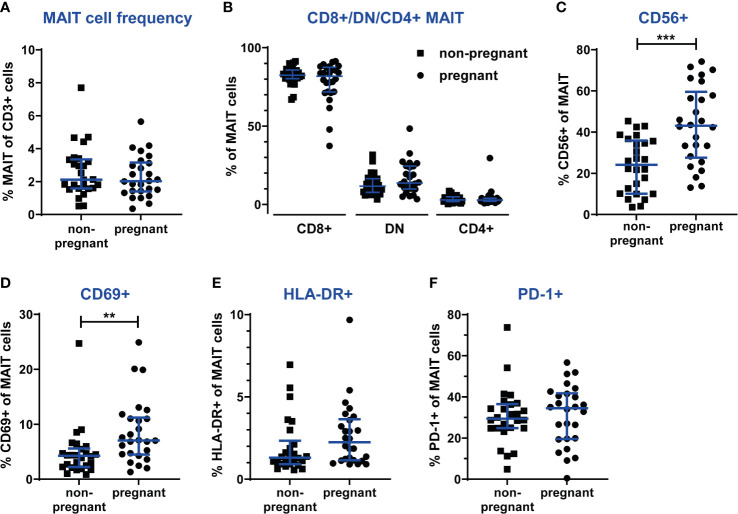
The frequency and phenotype of circulating MAIT cells are unaltered during the 3^rd^ trimester of pregnancy, but in pregnant women more MAIT cells express the activation marker CD69. PBMCs from women pregnant in the 3^rd^ trimester (n=26) and from non-pregnant, age-matched controls (n=26) were analyzed by flow cytometry for MAIT cells frequencies, MAIT cell subsets and the expression of activation markers. **(A)** MAIT cells numbers were analyzed using a gating strategy similar to the one shown in **Figure 1A** (live CD3^+^Vα7.2^+^CD161^hi^ cells). **(B)** Proportions of CD8^+^, CD4^+^ and double-negative (DN) subsets of MAIT cells. **(C)** Proportions of the CD56^+^ subset of MAIT cells. Cell surface expression of the activation markers **(D)** CD69, **(E)** HLA-DR and **(F)** PD-1. Bars show median and IQR and statistical comparisons were performed using Mann-Whitney test. ** p < 0.01, *** p < 0.001.

During the 3^rd^ trimester of pregnancy, the frequency of circulating MAIT cells was similar in non-pregnant and pregnant women (**Figure 4A**). Also, absolute MAIT cell counts in the blood samples did not differ between the groups (data not shown). Likewise, there was no difference in the frequency of CD8^+^, DN and CD4^+^ subsets of MAIT cells between the study groups (**Figure 4B**).

However, several phenotypic markers revealed alterations in circulating MAIT cells during pregnancy. First of all, the CD56^+^ subset of MAIT cells was significantly increased in pregnant women, from 24% (median) in non-pregnant to 43% in pregnant women (**Figure 4C**). This could reflect the general enhancement of innate immune functions during pregnancy. Furthermore, pregnant women exhibited a higher proportion of MAIT cells positive for the T cell activation marker CD69 (**Figure 4D**), while no differences between groups were observed for HLA-DR and PD-1 (**Figure 4E, F**).

Similar to MAIT cells, no differences were found for the frequencies of CD4^+^ and CD8^+^ T cells between pregnant and non-pregnant women (data not shown). However, analysis of the expression of CD69, HLA-DR and PD-1 revealed that in the circulation of pregnant women, significantly more CD4^+^ and CD8^+^ T cells expressed CD69 (**Supplementary Figure S5**), similar to MAIT cells (**Figure 4D**). Furthermore, more NK cells expressed CD69 and HLA-DR. For CD4^+^ T cells, no differences between groups were seen for HLA-DR expression, as well as for the expression of PD-1 by any lymphocyte subset (**Supplementary Figure S5**). The enhanced activation of NK cells again reflects the enhancement of innate responses during pregnancy.

### Circulating MAIT Cells Exhibit Stronger Functional Responses During Pregnancy

Activation of MAIT cells has been described to lead to a quick and strong reaction, which encompasses the production of proinflammatory cytokines and cytotoxic effector molecules. During pregnancy, especially during the 3^rd^ trimester, adaptive immune responses such as T and B cell activation are generally dampened, while innate responses are enhanced (1). Therefore, we asked whether MAIT cells, carrying characteristics of both innate cells and T cells, would show altered responses to stimulation during pregnancy. For stimulation we used both TCR-dependent (activating anti-CD3/CD28 antibodies, *E. coli*, group B streptococci), and TCR-independent (IL-12 and IL-18, influenza A virus) stimuli to activate PBMCs isolated from pregnant women in the 3^rd^ trimester, and from non-pregnant female controls.

First of all, we compared activation levels between circulating MAIT cells and CD4^+^ and CD8^+^ T cells. MAIT cells responded with the production of IFN*γ* to all tested stimuli, while CD4^+^ and CD8^+^ T cells in the same PBMC cultures did not respond or responded only slightly with IFN*γ* production (**Supplementary Figure S6A**). These findings show that MAIT cells reach higher levels of activation during the relatively short incubation period, confirming the innate nature of MAIT cells featuring rapid responses.

**Figure 5:**
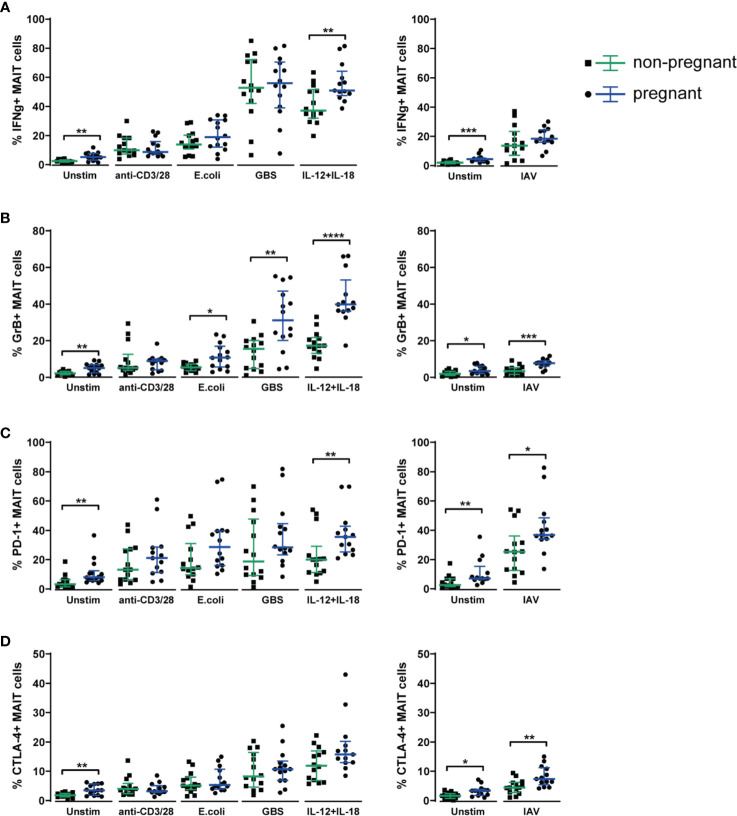
Circulating MAIT cells from 3^rd^ trimester pregnant women exhibit a stronger functional response upon stimulation with microbial and inflammatory stimuli compared with MAIT cells from non-pregnant women. PBMCs isolated from the blood of 3^rd^ trimester pregnant women (n=14) and from non-pregnant, age-matched controls (n=14) were stimulated overnight with plate-bound anti-CD3 and anti-CD28 antibodies, fixed *E coli*, fixed group B streptococci (GBS), a combination of IL-12 and IL-18, with influenza A virus (IAV) or left unstimulated (Unstim). IAV stimulations were carried out in a separate plate, together with a separate unstimulated control. MAIT cells in unstimulated and stimulated samples were analyzed for their expression of **(A)** IFN*γ*, **(B)** granzyme B (GrB), **(C)** PD-1 and **(D)** CTLA-4. For PD-1, cell surface expression was analyzed, whereas IFN*γ*, granzyme B and CTLA-4 were stained intracellularly. Results for CD69 from the same experiments are shown in **Supplementary Figure S6B**. Bars show median and IQR and statistical comparisons between the non-pregnant and pregnant group were performed using Mann-Whitney test. * p < 0.05, ** p < 0.01, *** p < 0.001, **** p < 0.0001. n = 13-14 for both groups.

Comparing MAIT cells responses in samples from pregnant and non-pregnant women, we observed increased IFN*γ* production in the pregnant group upon stimulation with combined IL-12 and IL-18, but also higher spontaneous IFN*γ* production in the unstimulated samples (**Figure 5A**), indicating that MAIT cells from pregnant women are primed to respond stronger to these innate cytokines, and that overnight cultivation, in the absence of stimulus, is enough to achieve a slight, yet significant increase in IFN*γ* production. Also, for all other readouts (granzyme B, PD-1, CTLA-4 and CD69), we observed higher expression in the unstimulated samples from the pregnant group (**Figures 5B–D** and **Supplementary Figure S6B**).

Regarding the production of granzyme B, we observed significantly more production in the pregnant group upon stimulation with the bacterial stimuli (*E. coli* and GBS), with IL-12 and IL-18 as well as with influenza A virus (**Figure 5B**). Likewise, the expression of the immune checkpoint markers PD-1 and CTLA-4, that often accompanies the activation of T cells, was significantly higher in the pregnant group upon stimulation with influenza A virus for both PD-1 and CTLA-4 (**Figures 5C, D**).

To summarize, the data from the *in vitro* stimulations indicate that during pregnancy, MAIT cells respond at least as strongly to stimulation as in the non-pregnant setting, and for several stimuli and markers, there were significantly increased responses in the pregnant group, especially for the TCR-independent stimuli, namely IL-12 and IL-18, and influenza A virus. Taken together, these findings indicate enhanced functional responses of MAIT cells during pregnancy.

## Discussion

Pregnancy constitutes an immunological challenge to the maternal immune system, with distinct alterations in both innate and adaptive immunity. MAIT as innate-like T cells feature both innate and adaptive properties, allowing them to react to microbial or inflammatory threats with the speed of innate immune cells, but with adaptive effector functions (8). Under the view of these dual characteristics of MAIT cells, we here investigated MAIT cells from the fetal-maternal interface and the blood circulation in healthy pregnancies.

During the 1^st^ trimester of pregnancy, we found MAIT cells to be relatively excluded from the decidua, while remaining MAIT cells exhibited an immunomodulatory phenotype compared with blood, for example by showing higher expression of immune checkpoint markers. During the 3^rd^ trimester of pregnancy, when systemic immunomodulation is most pronounced, MAIT cells showed an increased expression of the activation marker CD69, and after challenge with microbial and inflammatory stimuli they responded stronger than in the non-pregnant controls. This is the first study characterizing the functional response of MAIT cells during the 3^rd^ trimester of pregnancy, and the first study revealing a detailed insight into frequency and functional immune phenotype of MAIT cells in the decidua during early pregnancy.

In the present study we confirm that T cells are generally excluded from the decidua (1, 2), and we also reveal that MAIT cells are decreased to an even higher degree, which could reflect protective mechanisms to limit the presence of these reactive and potentially highly inflammatory cells. Along the same lines, the expression of PD-1 and CTLA-4 by the remaining decidual MAIT cells combined with the almost complete absence of granzyme B expression may suggest that these cells are subjected to immunomodulatory mechanisms and themselves could exert immunomodulatory functions, although further studies are needed to prove this. The increased expression of HLA-DR, PD-1 and CTLA-4 by decidual MAIT cells is similar to MAIT cells in other tissues, such as the buccal mucosa and the gut (39, 40), and at steady state, neither circulating nor tissue-resident MAIT cells express granzyme B (8). Furthermore, the alteration in subset composition of decidual MAIT cells provides evidence for immunomodulation of MAIT cells at the fetal-maternal interface. Thus, the decrease in the CD56^+^ MAIT subset, which has been linked to enhanced innate characteristics including stronger responses to innate cytokines (21) and to more severe COVID-19 disease (35), could protect from excessive inflammatory responses. Also, the CD4^+^ MAIT cell subset, which we found to be increased in the decidua, has been described to exert more immunomodulatory action as compared with the cytotoxic CD8^+^ and DN subsets (18). However, the exact identity of the CD4^+^ MAIT subset is not settled, and CD4^+^Vα7.2^+^CD161^+^ cells may be unable to recognize the MR1 tetramer that defines MAIT cells (19). In our study, these cells could therefore recognize non-MAIT antigens, and could represent conventional T cells restricted to classical MHC molecules and other innate-like T cells such as the CD4^+^ germline-encoded mycolyl lipid-reactive (GEM) T cells that use the Vα7.2 segment in their TCRs (41). Hence, the implication of differences in CD8/DN/CD4 subsets in the decidua should be interpreted with caution.

Taken together, our findings related to the fetal-maternal interface indicate consistent and broad phenotypic alterations in decidual MAIT cells, with the aim to preserve immune tolerance. Besides MAIT cells, also non-MAIT CD4^+^ and CD8^+^ T cells are subjected to immunomodulation, as observed by us and others through the increased numbers of T cells expressing PD-1 and CTLA-4, and decreased proportion of granzyme B-expressing NK and CD8^+^ T cells (42). These similarities between MAIT cells, non-MAIT T cells and NK cells suggest shared mechanisms of immunomodulation for all decidua-resident and potentially proinflammatory lymphocytes. The exact mechanisms behind this modulation are unclear, but most likely linked to the secretion of immune dampening factors such as IL-10 and M-CSF produced by macrophages, trophoblast cells and stromal cells, and occurs under the influence of pregnancy hormones and other placental factors (1, 43–45).

Despite their immunomodulatory phenotype and low abundance, we speculate that it is still possible that 1^st^ trimester decidual MAIT cells, given their role in other tissues (8), could switch phenotype on demand or be quickly recruited from blood, and thereby contribute to defense from infection in a manner independent of conventional peptide-antigen presentation by classical MHC molecules. Due to low decidual MAIT cell numbers and our prioritization of other phenotypic analyses, we were unable to investigate whether MAIT cells exhibit characteristics of tissue-resident cells. A recent study focusing on endometrial MAIT cells reported that decidual MAIT cells (n=3) expressed markers of tissue residency (26). Furthermore, we did not investigate functional responses of MAIT cells in 1^st^ trimester decidua. Studies on 3^rd^ trimester decidua showed MAIT cells to respond to bacterial challenge with the production of IFNγ, granzyme B and perforin, indicating potential involvement in antibacterial defenses (22, 24, 32). Given their tissue repair potential (8), also the transcriptional activity of MAIT cells would be relevant to investigate as well as their role in endometrial tissue remodeling during decidualization (in similarity to uterine NK cells). Furthermore, the exact factors that induce immunomodulatory phenotypes in MAIT cells are elusive, and also MAIT cells themselves have been shown to secrete central factors of decidual immunomodulation, IL-10, TGF-β and M-CSF (8, 46), and could therefore contribute to maintenance of tolerance at the fetal-maternal interface.

One potential limitation for the analysis of decidual immune cells is that the participating women had all received misoprostol, which was in general administered three hours before the planned procedure. It has been suggested that misoprostol could inhibit innate immunity, however, this has mainly been shown in rats (47) under artificial conditions (48), while in humans no such effects were noted (49). This notion is supported by our previous findings (50) of no differences in gene expression of decidual macrophages when comparing exposed and non-exposed women, although this was based on few observations (n=4 *versus* n=7). Taken together, we consider it less likely that Misoprostol would have an impact on our findings, although we cannot exclude this possibility.

Although MAIT cells are mainly tissue-resident cells, circulating MAIT cells and their functional responses have been investigated in many conditions and diseases, but pregnancy has so far been neglected. In our cohort of healthy 3^rd^ trimester pregnant women and non-pregnant controls, pregnancy was associated with increased expression of the activation marker CD69 on MAIT cells. MAIT cells could be part of the ongoing enhancement and activation of innate immunity during pregnancy, such as increased activity of the complement system and elevated cellular numbers, activation states and proinflammatory functions of monocytes and neutrophils (27). The same phenomenon could underlie the observed “priming” of MAIT cells to mount significantly increased expression of all investigated markers in the unstimulated samples in the pregnant group. It remains to be elucidated whether MAIT cells themselves are primed to respond stronger, or whether the autoactivation of MAIT cells in unstimulated samples is due to enhanced or ongoing immune activation of innate cells in the PBMC cultures. Besides that, also the increase in the CD56^+^ subset of circulating MAIT cells in pregnant women could reflect or be involved in enhanced innate responses during pregnancy.

Although demonstrated for all markers, including the immune checkpoint markers, the increase in systemic functional MAIT cell responses in pregnant women was most pronounced for granzyme B, suggesting a skewing of MAIT functions towards antimicrobial defenses. Indeed, another study found MAIT cells numbers at term pregnancy to be decreased in the circulation, coinciding with accumulation in the intervillous space and increase in cytotoxicity, as compared with their circulating counterparts (22, 23). In our study, we investigated MAIT cell numbers and functions in blood during gestational week 36 instead of term pregnancy, and at this time point failed to show differences in circulating MAIT cell numbers. Furthermore, the elevated granzyme B production observed in the pregnant group as compared to the non-pregnant group upon bacterial and viral challenge was not reflected in the IFNγ production, except for the inflammatory stimulus of combined IL-12 and IL-18. This elevated granzyme B production observed in the pregnant group as compared to the non-pregnant group upon bacterial and viral challenge was not reflected in the IFN*γ* production. This could be a mechanism to alter the inflammatory response, similar to the reported decreased IFN*γ* responses by CD4^+^ T cells during pregnancy (51), and to avoid excessive inflammation.

Overall, the expression of CD69, the increase in the CD56^+^ MAIT subset and the stronger response of circulating MAIT cells to microbial and inflammatory stimuli could reflect compensatory mechanisms for the dampened adaptive responses during pregnancy, which are most apparent during the 3^rd^ trimester. MAIT cells, situated at the intersection of innate and adaptive responses, are suitable to be “on stand-by” to react quickly when needed, for example in the case of microbial threats. Despite these and other compensatory mechanisms, immune responses during pregnancy are not always sufficient, which manifests in increased susceptibility to certain bacterial infections, and in more severe viral diseases (5, 27). The increased risk of influenza infection in pregnant women has been attributed to decreased IFN*γ* production in mice (52) and *ex vivo* in human PBMCs (53), whereas another study did not find any differences in intracellular IFN*γ* upon influenza stimulation in CD4^+^ and CD8^+^ T cells (54). We here demonstrated stronger MAIT cells responses *in vitro* to influenza A virus infection not for IFN*γ* but for granzyme B, PD-1 and CTLA-4. While MAIT cells have been perceived to generally protect from influenza infection (55), their increased activation during pregnancy could be associated with a more severe disease course. In a similar manner, MAIT cells could be contributing to a more severe COVID-19 disease in pregnant women (56).

Besides an increased response to influenza A virus, MAIT cells from pregnant women responded strongly to a hemolytic strain of GBS. In general, vaginal colonization with GBS is common, and ascending infections and invasion of reproductive tissues are associated with preterm labor and preterm birth, which has been linked to the presence of certain virulence factors such as hemolytic pigment (28). In HIV-positive pregnant women, who have a higher risk of delivering preterm, MAIT cell subsets have been shown to be altered in the preterm group compared with term delivery, exhibiting an increased proportion of CD8^+^ MAIT cells (57). In the decidua, MAIT cells could be involved in early defenses against GBS, as well as in the arising inflammation leading to massive neutrophil recruitment (28). Besides a potential involvement in preterm birth, MAIT cells have also been studied in early-onset preeclampsia, where circulating MAIT cells were found to be decreased and phenotypically altered, with lowered PD-1 and increased CD69 and perforin expression (58). Overall, more detailed characterizations and studies on the potential recruitment of MAIT cells to and activation in the decidua in pregnancy complications are so far missing, and their exact role in preterm birth and preeclampsia remains to be settled.

To conclude, the findings from our study indicate dual roles for MAIT cells during pregnancy, with a seemingly well-adapted ability to balance the requirements of immune tolerance in parallel with maintained antimicrobial defenses. During early pregnancy, when establishment of local tolerance in the decidua is crucial for a successful and uncomplicated pregnancy, MAIT cells are restricted in the decidua, both numerically and phenotypically. During the 3^rd^ trimester of pregnancy, when systemic immunomodulation is most pronounced, MAIT cells show enhanced functional responses, indicative of antimicrobial defenses that compensate for the weakening of the adaptive T cell responses. Dysregulation of MAIT cells could lead to detrimental inflammation and preterm loss of immune tolerance, which have been suggested as underlying mechanisms for preterm labor and preeclampsia. Future studies should hence address the potential involvement of MAIT cells in pregnancy complications.

## Data Availability Statement

The raw data supporting the conclusions of this article will be made available by the authors, without undue reservation.

## Ethics Statement

The studies involving human participants were reviewed and approved by regional ethics review board in Linköping. The patients/participants provided their written informed consent to participate in this study.

## Author Contributions

JR, ML and JE conceived the original research idea. JR, RL, GB, ML and JE designed the study. JR, RL and SV designed and conducted experiments and analyses. JR, RL, SV, ML and JE interpreted the results. GB and JR recruited study volunteers and reviewed patient journals. JR and JE wrote the manuscript. All authors contributed to the article and approved the submitted version.

## Funding

This work has been supported by: MIIC (Medical Infection and Inflammation Center, Linköping University and Region Östergötland) seed grant (ML and JE) and MIIC postdoc grant (JE), FORSS (Medical Research Council of Southeast Sweden, ML FORSS-657691, FORSS-751571 and FORSS-850071), the Swedish Research Council (ML 2017-01091, JE 2018-02776) and Linköping University Hospital Research Fund (ML and JE).

## Conflict of Interest

The authors declare that the research was conducted in the absence of any commercial or financial relationships that could be construed as a potential conflict of interest.

## Publisher’s Note

All claims expressed in this article are solely those of the authors and do not necessarily represent those of their affiliated organizations, or those of the publisher, the editors and the reviewers. Any product that may be evaluated in this article, or claim that may be made by its manufacturer, is not guaranteed or endorsed by the publisher.
