# Supplementary material for: MAIT Cells Balance the Requirements for Immune Tolerance and Anti-Microbial Defense During Pregnancy
Source: Front Immunol. 2021 Aug 9;12:718168. doi: 10.3389/fimmu.2021.718168 (PMC8420809; doi:10.3389/fimmu.2021.718168)
Supplement: Supplementary file 1 [file DataSheet_1.pdf]

# Supplementary Material

## Supplementary Figures and Supplementary Table

## Supplementary Figure S1

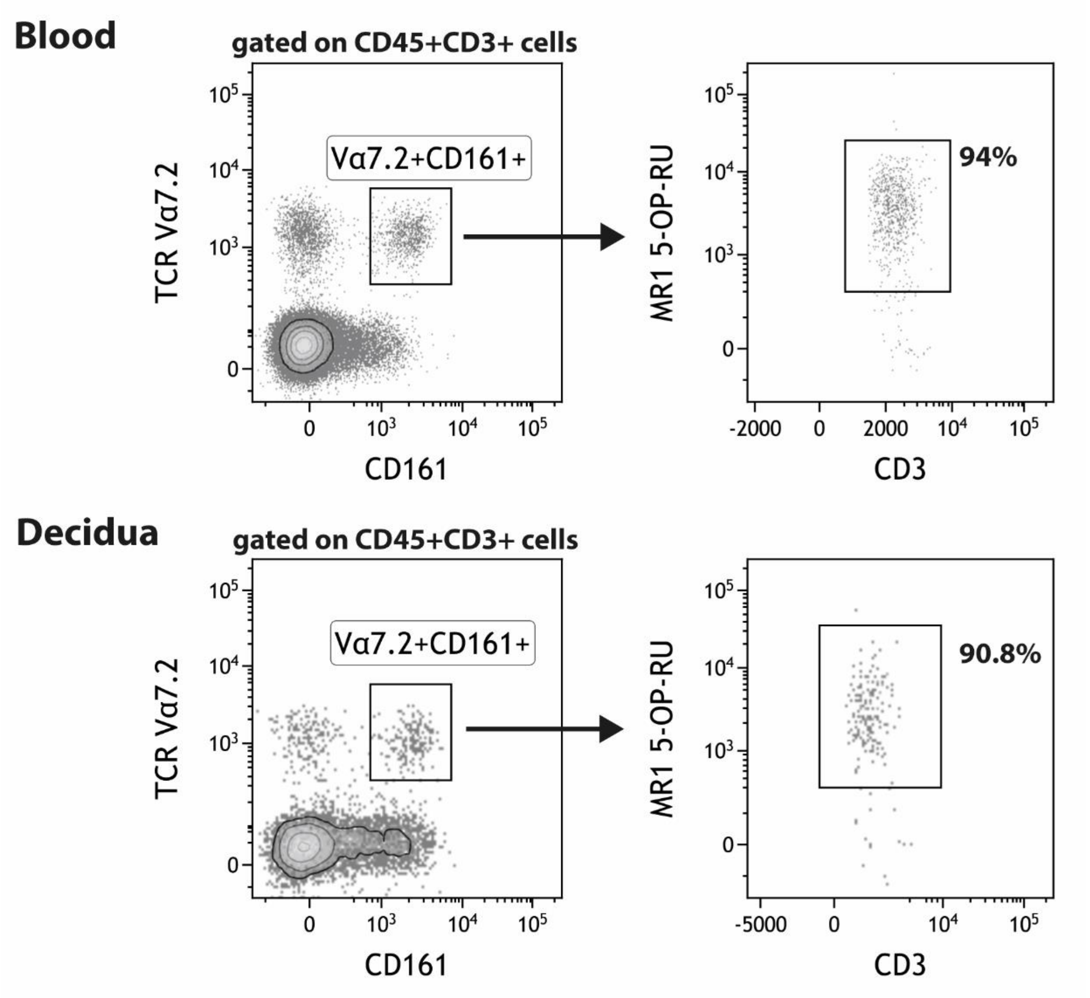


**Supplementary Figure S1: The majority of Vα7.2$^{+}$CD161$^{+}$ MAIT cells are positive for the MR1 tetramer**

MR1 tetramer staining of paired blood and decidua samples was carried out in combination with staining for the surface markers CD45, CD3, Vα7.2 and CD161. Vα7.2$^{+}$CD161$^{+}$ MAIT cells were gated among CD45$^{+}$CD3$^{+}$ cells (left panels), and in the next step (right panels) analyzed for binding of the MR1 5-OP-RU tetramer. The MR1 6-FP tetramer was used as a negative control and to define the gate for MR1 5OP-RU-positive cells (not shown).

## Supplementary Figure S2

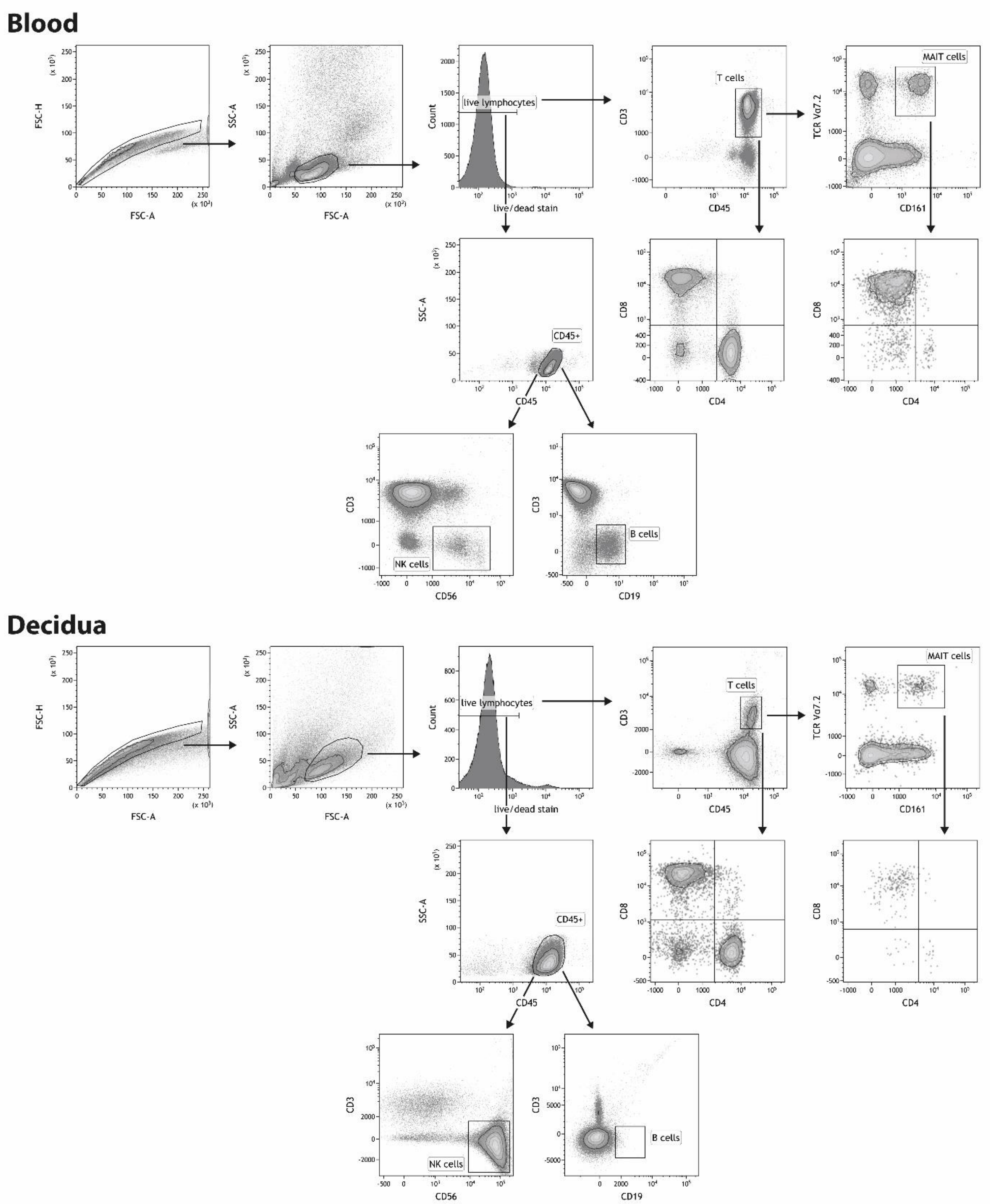


**Supplementary Figure S2: Gating strategies for lymphocyte subsets in blood and decidua**

Peripheral blood mononuclear cells (PBMCs) and decidual cells were isolated from blood and 1st trimester decidual tissue, respectively, from the same donors, and analyzed by flow cytometry. Gating strategy for T cells, MAIT cells, NK cells and B cells, as well as for $CD8^{+}$, $CD4^{+}$ and double-negative T cells and MAIT cells are shown for one representative paired sample.

## Supplementary Figure S3:

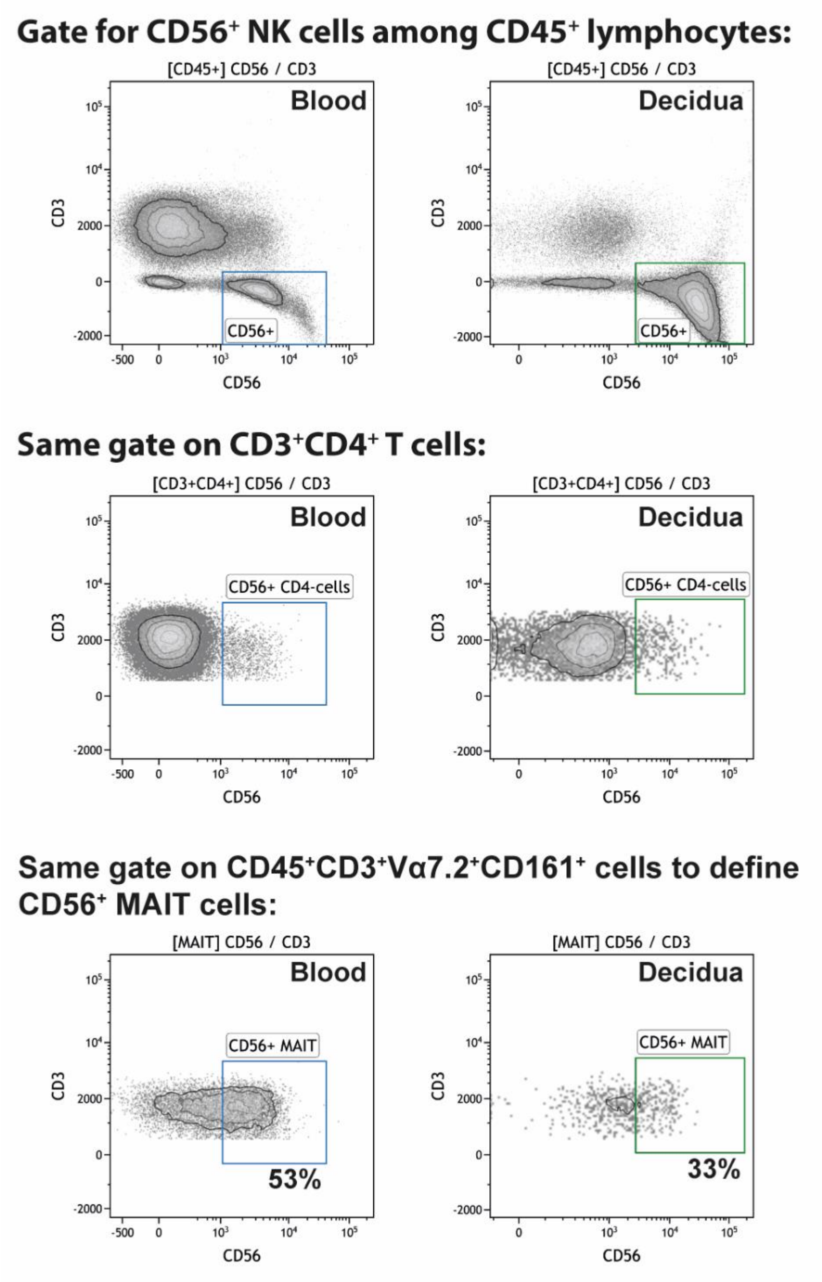


**Supplementary Figure S3: Gating strategy for CD56$^+$ MAIT cells in blood and decidua**

Peripheral blood mononuclear cells (PBMCs) and decidual cells were isolated from blood and 1$^{st}$ trimester decidual tissue, respectively, from the same donors, and analyzed by flow cytometry. CD56$^+$ lymphocytes (NK cells, upper panels) and CD4$^+$ T cells (middle panels, chosen because they are mostly CD56$^-$) were used to set the gate for CD56$^+$ MAIT cells (lower panels). Percentages of CD56$^+$ MAIT cells are presented in **Figure 2C**.

## Supplementary Figure S4

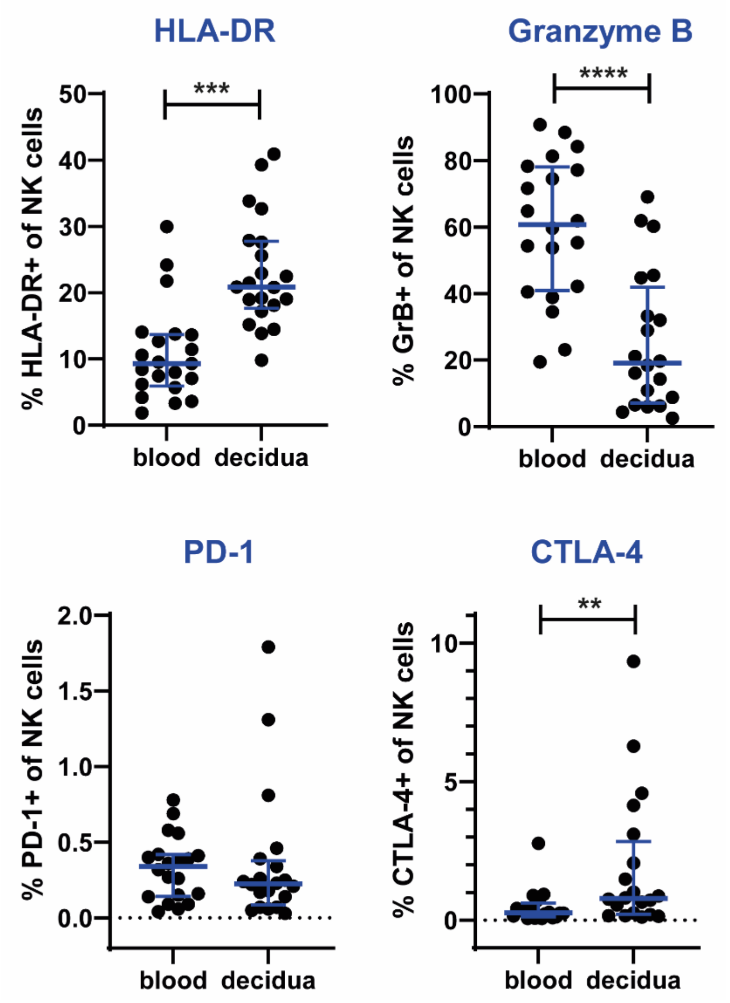


**Supplementary Figure S4: Expression of phenotypic markers by circulating and decidual NK cells**

Peripheral blood mononuclear cells (PBMCs) and decidual cells were isolated from blood and decidual tissue, respectively, from the same donors, and analyzed by flow cytometry for expression of HLA-DR, granzyme B, PD-1 and CTLA-4 by NK cells. HLA-DR and PD-1 were analyzed on the cell surface, whereas granzyme B and CTLA-4 were stained intracellularly. Bars show median and interquartile range (IQR) from 20-21 donors. Statistical comparisons were performed with Wilcoxon signed-rank test. ** p<0.01, ***p<0.001, **** p<0.0001.

## Supplementary Figure S5

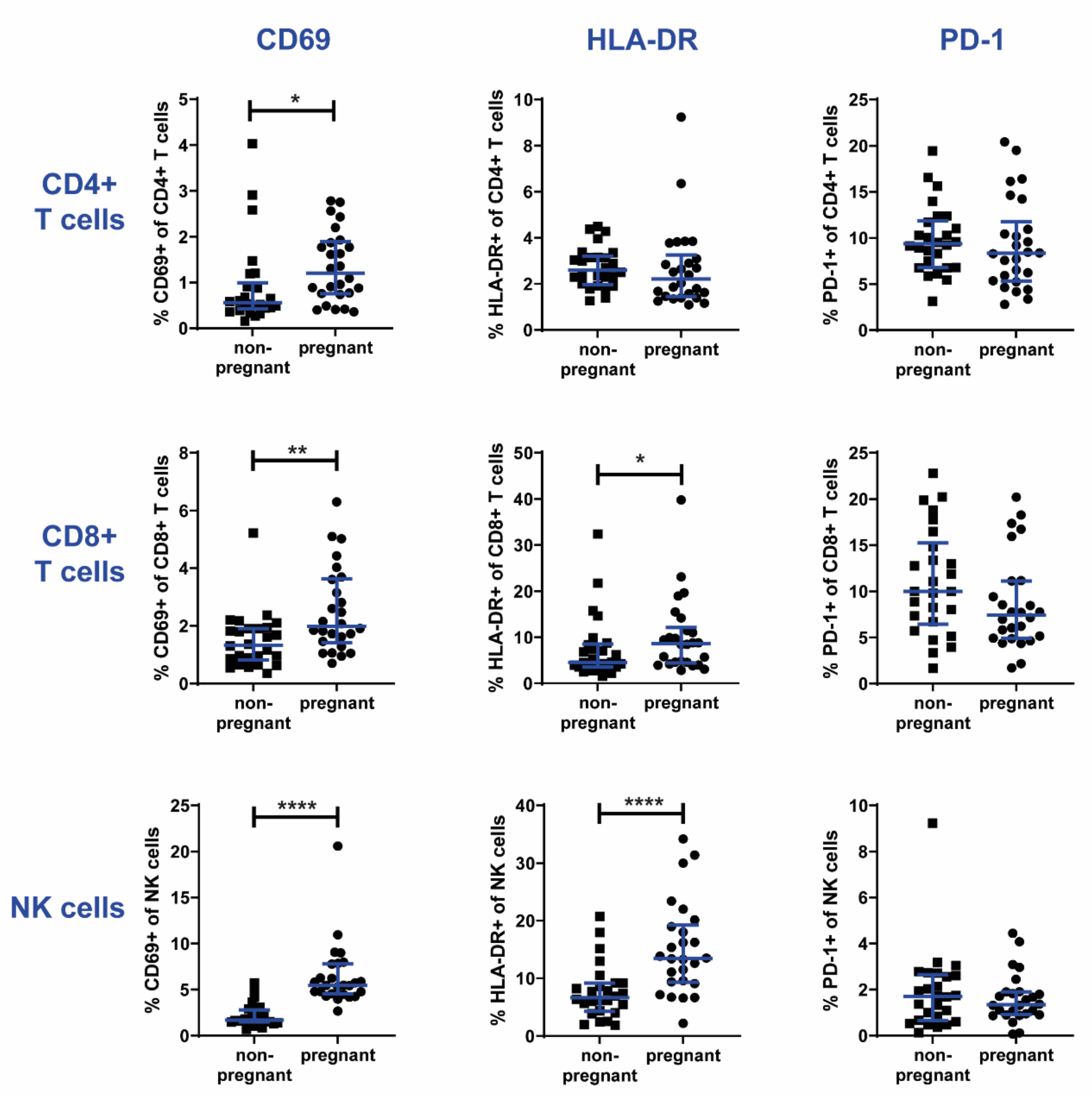


**Supplementary Figure S5: Expression of phenotypic markers by circulating CD4$^+$ and CD8$^+$ T cells and NK cells in the 3$^{rd}$ trimester of pregnancy**

Peripheral blood mononuclear cells (PBMCs) from women pregnant in the 3$^{rd}$ trimester (n=26) and from non-pregnant, age-matched controls (n=26) were analyzed by flow cytometry for the expression of CD69, HLA-DR and PD-1 by CD4$^+$ and CD8$^+$ T cells, as well as by NK cells. Bars show median and IQR and statistical comparisons were performed using Mann-Whitney test. *p<0.05, **p<0.01, **** p<0.0001.

## Supplementary Figure S6

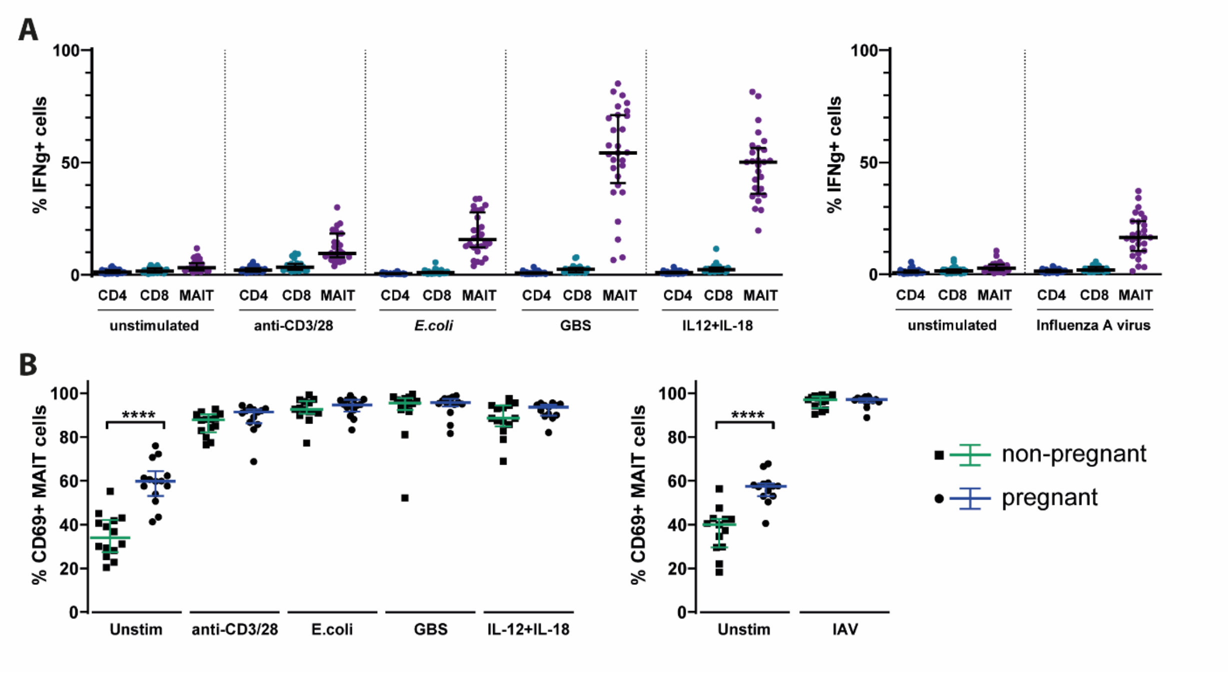


**Supplementary Figure S6: MAIT cells exhibit a stronger functional response to microbial and inflammatory stimuli than CD4⁺ and CD8⁺ T cells in the same cultures, and a high proportion of stimulated MAIT cells expresses the activation marker CD69**

Peripheral blood mononuclear cells (PBMCs) isolated from the blood of 3rd trimester pregnant women and from non-pregnant, age-matched controls were stimulated overnight with plate-bound anti-CD3 and anti-CD28 antibodies, fixed *E. coli*, fixed group B streptococci (GBS), a combination of IL-12 and IL-18 (left panels), and with influenza A virus (IAV, right panels) or left unstimulated (Unstim). IAV stimulations were carried out in a separate plate, together with a separate unstimulated control. **(A)** shows the expression of IFNγ by $CD4^+$ and $CD8^+$ T cells and MAIT cells in unstimulated and stimulated samples. For this graph, the data from the pregnant and non-pregnant women are combined and presented as a total; n=27-28. Bars show median and IQR. **(B)** shows the expression of CD69 upon stimulation, for non-pregnant and pregnant women. The data for expression of IFNγ, granzyme B, PD-1 and CTLA-4 in the same experiments are shown in **Figure 5**. N=13-14 for both groups. Bars show median and IQR from 20-21 donors. Statistical comparisons in (B) were performed with Mann-Whitney test. **** p<0.0001.

## Supplementary Table S1. Flow cytometry antibodies and panels used in this study

x indicates that the antibody was used for surface staining on intact cells. **xx** indicates that this marker was stained intracellularly, after staining with antibodies for surface markers (x), fixation and permeabilization of the cells. PBMC, peripheral blood mononuclear cells.

| | | | | | 1st trimester samples; PBMCs and decidua | | | | 3rd trimester PBMCs | |
|---|---|---|---|---|---|---|---|---|---|---|
| **Marker/Antibody** | **Clone** | **Host species and isotype** | **Fluorochrome** | **Manufacturer** | **Surface markers** | **Surface and intracellular markers** | **B cell panel** | **Tetramer staining** | **Phenotyping of freshly isolated PBMCs** | **Stimulations of PBMCs** |
| | | | | | **Figure 1-3, S2, S3, S4** | **Figure 3, S4** | **Figure 1, S2** | **Figure S1** | **Figure 4, S5** | **Figure 5, S6** |
| Aqua LIVE/DEAD stain | NA | | | Molecular Probes | x | x | x | x | x | x |
| CD45 | 2D1 | mouse IgG1 | APC-H7 | BD | x | x | x | x | | |
| CD3 | UCHT1 | mouse IgG1 | Alexa Fluor 700 | BD | x | x | x | x | x | x |
| CD4 | RPA-T4 | mouse IgG1 | PerCP-Cy5.5 | BD | x | x | | x | x | x |
| CD8 | SK1 | mouse IgG1 | BV605 | BD | x | x | | x | | |
| CD8 | SK1 | mouse IgG1 | APC-H7 | BD | | | | | x | x |
| CD56 | NCAM16.2 | mouse IgG2b | BV711 | BD | x | x | x | x | x | x |
| TCR Vα7.2 | 3C10 | mouse IgG1 | PE | Biolegend | x | x | | x | x | x |
| CD161 | DX12 | mouse IgG1 | APC | BD | x | x | | x | x | x |
| isotype control | 27-35 | mouse IgG2b | FITC | BD | x | | | | | |
| HLA-DR | L243 | mouse IgG2a | FITC | BD | x | | | | x | |
| isotype control | MOPC-21 | mouse IgG1 | PE-Cy7 | BD | x | | | | | |
| PD-1 | EH12.1 | mouse IgG1 | PE-Cy7 | BD | x | | | | | |
| isotype control | G155-178 | mouse IgG2a | BV421 | BD | | **xx** | | | | |
| CTLA-4 | BNI3 | mouse IgG2a | BV421 | BD | | **xx** | | | | |
| isotype control | MOPC-21 | mouse IgG1 | FITC | BD | | **xx** | | | | |
| Granzyme B | GB11 | mouse IgG1 | FITC | BD | | **xx** | | | | **xx** |
| isotype control | X40 | mouse IgG1 | BV605 | BD | | | | | x | |
| PD-1 | EH12.1 | mouse IgG1 | BV605 | BD | | | | | x | x |
| CD19 | SJ25C1 | mouse IgG1 | PerCP | BD | | | x | | | |
| MR1/6-FP | Tetramer | | PE | NIH Tetramer Core Facility | | | | x | | |
| MR1/5-OP-RU | Tetramer | | PE | NIH Tetramer Core Facility | | | | x | | |
| IFNγ | 4S.B3 | mouse IgG1 | BV421 | BD | | | | | | **xx** |
| CD69 | FN50 | mouse IgG1 | BV786 | BD | | | | | | x |
| CTLA-4 | BNI3 | mouse IgG2a | PE-Cy7 | Biolegend | | | | | | **xx** |
